# Identification of risk factors for human immunodeficiency virus-1 infection in Khyber Pakhtunkhwa population: A case control study

**DOI:** 10.12669/pjms.35.5.258

**Published:** 2019

**Authors:** Jamila Haider, Ghosia Lutfullah, Irshad ur Rehman, Irfan Khattak

**Affiliations:** 1 Jamila Haider, BS. PhD. Scholar, Centre of Biotechnology & Microbiology, University of Peshawar, Peshawar, Pakistan; 2 Prof. Dr. Ghosia Lutfullah, M. Phil, PhD. Ex. Director, Centre of Biotechnology & Microbiology, University of Peshawar, Peshawar, Pakistan; 3 Irshad ur Rehman, M. Phil, PhD. Assistant Professor, Centre of Biotechnology & Microbiology, University of Peshawar, Peshawar, Pakistan; 4 Dr. Irfan Khattak, DVM, PhD. Assistant Professor, College of Veterinary Sciences & Animal Husbandry, Abdul Wali Khan University, Mardan, Pakistan

**Keywords:** Risk Factors, Human Immunodeficiency Virus-1 (HIV), Injection drug users (IDUs), Blood transfusion, tattooing, Khyber Pakhtunkhwa (KP)

## Abstract

**Objectives:**

The present study aims to identify the risk factors for Human Immunodeficiency Virus-1(HIV-1) infection in Khyber Pakhtunkhwa (KP) population by comparing HIV-antibody positive cases with HIV-antibody-negative controls.

**Methods:**

The study was designed at the Family Care Centre (FCC), Hayatabad Medical Centre (HMC) Peshawar from February 2015 to December 2016. A total of 280 individuals were selected randomly for the study as cases and control. Data was collected on a structured questionnaire with informed oral consent. The collected data was analysed statistically using SPSS version 20.

**Results:**

Out of 280 individuals, 56% were males, 44% were females, and 53.21% belonged to the urban areas. The literacy rate was 48.6%, and 75.4% were married. The statistical analysis of risk factors revealed the following factors as of significance value (p < 0.05). Family history of HIV (OR = 9.46), spouse status of HIV (OR=22.22), injection drug users (IDUs), migrants (OR=2.234), use of therapeutic injections (OR= 2.791), employment (OR=2.545), male gender (OR=2.35), tattooing (OR=7.667) and history of blood transfusion (OR= 2.69).

**Conclusion:**

The present study revealed spouse status of HIV, tattooing, migrants, IDUs, use of therapeutic injections, history of blood transfusion, male gender and employment as significant risk factors for HIV infection in the population of KP.

## INTRODUCTION

The characteristics feature of HIV is the continued deprivation of the human immune system, a condition better known as the acquired immunodeficiency syndrome (AIDS).^1^ The infection is transmitted by semen, vaginal fluid, breast milk, pre-ejaculate and blood transfer.^2^ The virus is present as free particles in these body fluids. After entry, the virus infects cells of the immune system such as macrophages, dendritic cells and CD4+ T cells.^3^ The CD4+ cell acts as a host for replication of HIV and infects other cells. As a result, there is a fall of CD4+ cells in the body and failure of the immune system. This decrease of CD4+ cells is used as a marker for the development of AIDS.^4^

Since last 30 years, the HIV/AIDS epidemic has raised as one of the major challenges for the world, starting from a relatively small problem in the 1980s to one of the leading cause of mortality and burden over the last decade.^4^-^6^ Globally, since the start of the epidemic 78 million [69.5 million–87.6million] people have become infected with 2.1 million [1.8 million–2.0million] newly infected cases in the year 2015 and 35 million [29.6 million–40.8 million] people have died due to AIDS-related illnesses.^7^

In 1987, the first case of HIV/AIDs was reported in Pakistan. An estimated 91,340 people (range 61,000–128,000) were living with HIV as of December 2014, and another 15,606 people (range 7000–33,000) became newly infected with HIV in that year.^8^ However, the reliability of existing data is limited and real rates of HIV infection in Pakistan is much higher than what the official report suggested. The main reason for under-reporting is the social stigma, the threat of social isolation linked to this infection and unawareness of the people regarding their state of illness.^9^

Although, Pakistan currently shows a low prevalence of HIV however, there are concerns for a widespread epidemic mainly due to presence of numerous socio-economic conditions, unsafe high-risk practices, limited knowledge about HIV/AIDS and its geographic location among HIV/AIDS high-risk countries; China in the north, India on the east, Afghanistan on the west and the only low HIV prevalence country is Iran on south. In Pakistan, the major modes of HIV transmissions are sexual, infectious blood and IDUs. Another factor that poses serious threats to the Pakistani population is a common delusion that being Muslim, HIV cannot be contracted. Such prudence stresses on constant monitoring and preventive interventions to control the spread of HIV.^10^

Currently, there is no known cure for HIV infection. However, the use of antiretroviral treatment can slow down the course of the disease decreasing the risk of death and complications from the disease. The prevention of infection should be the aim in all at risk communities. Knowledge of the prevalent risk factors is of paramount importance for developing plans to control the incidence of infection.^11^ Therefore; the present study was designed to identify the risk factors of HIV-1 infection in the KP population by comparing HIV-antibody positive cases with HIV antibody-negative controls.

## METHODS

The current case-control study was conducted at FCC, HMC Peshawar during the year 2015-2016. Currently, this is the main centre in KP providing the facility of HIV testing, antiretroviral (ARV) treatment, psychological counselling and awareness.

The cases of the study included the patients visiting the FCC. All of them were HIV-antibody positive tested by immuno-chromatography for HIV1/2 provided by the National AIDS Control Program (NACP) further confirmed by Enzyme Linked Immuno Sorbent Assay (ELISA) and PCR. They are referred from government and private sector hospitals as well from Non-Government Organizations (NGOs).Cases included both prevalent as well as incident seroconversion. The controls were HIV-antibody negative individuals selected from healthy blood donors of the blood bank, HMC Peshawar. A total of 140 controls and 140 cases of both gender were selected randomly for the study. The participants were informed about the study and informed oral consent was taken before collecting the information.

The data was collected on a structured questionnaire that consists of personal/demographic data, family history of HIV and a history of risk factors of acquiring the infection. In marital status, married is defined as ever married irrespective of the current status of divorce or widow. The study was reviewed and approved by the ethical committee of the Department of Pharmacy, University of Peshawar.

Statistical analysis of the data was done using SPSS version 20. The odds ratio, confidence interval and percentage were calculated along with bivariate analysis.

## RESULTS

The present study interviewed a total of 280 participants among them males were in higher proportion (56.0%) as compared to females (44.0%) belonging mostly to urban areas (53%). 48.6% were literate having education level from under primary to Masters (data not shown here). A large proportion was married (75.4%) as compared to unmarried. General characteristics of study subjects are represented in Table-I.

**Table I:** General characteristics of study subjects.

Characteristics	Cases N=140	Control N=140	Overall Percentage %
Male	93	64	56.0%
Female	47	76	44.0%
Illiterate	78	66	51.4%
Literate	62	74	48.6%
Married	111	100	75.4%
Unmarried	29	40	24.6%
Rural	67	64	46.79%
Urban	73	76	53.21%
***Age(yrs.)***			
<35	82	92	62.1%
>35	58	48	37.9%

Demographic risk factors for HIV infection are shown in Table-II. Age, gender, residence, education, employment status and marital status as risk factors for HIV infection were evaluated. It was observed that males were two times more likely to be infected as compared to females with a confidence interval of 95%(1.449-3.81) and a p-value of 0.001. Another risk factor found in demographic characters was the state of being employed or unemployed. The employment status reached the level of significance p-value 0.0001.

**Table II:** Demographic risk factors for HIV-1 infection in cases as compared with control.

Factors		Cases (N=140)	Control (N=140)	Odds ratio	CI	p-value

		N (%)	N (%)			
Age	<35 years	82 (58.57)	92 (65.71)	0.73	0.454-1.198	0.218
	>35 years	58 (41.43)	48 (34.29)			
Life Style	Rural	67 (47.86)	64 (45.71)	1.09	0.681-1.743	0.719
	Urban	73 (52.14)	76 (54.29)			
Gender	Male	93 (66.43)	64 (45.71)	2.35	1.449-3.81	0.001
	Female	47 (33.57)	76 (54.29)			
Marital Status	Married	111 (79.29)	100 (71.43)	1.53	0.884-2.651	0.127
	Unmarried	29 (20.71)	40 (28.57)			
Education Status	Illiterate	78 (55.71)	66 (47.14)	1.41	0.881-2.258	0.709
	Literate	62 (44.29)	74 (52.86)			
Employment Status	Employed	82 (58.57)	50 (35.71)	2.54	1.571-4.122	0.0001
	Unemployed	58 (41.43)	90 (64.29)			

Sexual and non-sexual related factors for HIV infection are expressed in Table-III. Non-sexual related variables included the previous history of different medical procedures, Injection drug use, piercing activities, sharing of shaving instruments and history of abroad travelling (as Pakistan is bordered by high-risk HIV countries). On the part of sexual activities, a detailed history of sexual behaviour/sexual partners was not available because most of the study participants were reluctant in giving such details.

**Table III:** Sexual and non-sexual related factors for HIV-1 infection in cases (N=140) as compared with Control (N=140).

Factors	Cases N (%)	Control N (%)	Odds ratio	CI	p-value
***Family History of HIV***					
Yes	59 (42.15)	10(7.14)	9.469	4.584-19.56	0.001
No	81 (57.85)	130(92.86)
***Relationship***					
Spouse	50 (84.75)	2(20)	22.22	4.043-122.2	0.001
Others	9 (15.25)	8(80)
***Surgical operation***					
Yes	35 (25.0)	25(17.86)	1.533	0.86-2.73	0.14
No	105 (75.0)	115(82.14)
***Dental treatment***					
Yes	67 (47.86)	55(39.29)	1.418	0.882-2.279	0.148
No	73 (52.14)	85(60.71)
***Blood transfusion***					
Yes	24 (17.14)	10(7.14)	2.69	1.234-5.861	0.010
No	116(82.86)	130(92.86)
***Therapeutic Injection use***					
Yes	92(65.7)	57(40.71)	2.791	1.718-4.534	0.001
No	48(34.3)	83(59.29)
***IDUs***					
Yes	14(10)	0(0)	-	-	0.001
No	126(90)	140(100)
***Tattooing***					
Yes	14(10)	2(1.42)	7.667	1.709-34.4	0.002
No	126(90)	138(98.58)			
***Acupuncture***					
Yes	7(5)	0 (0)	-	-	0.07
No	133(95)	140(100)			
***Sharing of shaving instrument***					
Yes	18(19.35)	10(15.63)	1.294	0.55-3.132	0.5
No	75(80.65)	54(84.37)			
***Migrants***					
Yes	53(37.86)	30(21.43)	2.234	1.316-3.79	0.002
No	87(62.14)	110(78.57)			
***MSW***					
Yes	3 (2.14)	0(0)	-	-	0.082
No	137(97.86)	140 (100)			

The history of HIV in blood relations and friends (categorized as others) and spouse were higher in cases as compared to controls. Statistical analysis showed that study subjects with history of HIV were nine times more prone to infection [p-value 0.001] as compared to those having no history of HIV.

Further analysis of the family history of HIV revealed that most of the spouses (84.75%) were infected with HIV as compared to others. The odds ratio was 22.22 with 95%CI [4.043- 122.2] that means the odds ratio of spouse with HIV is 22 times in cases as compared to controls with p-value of 0.001.

Only three men and no female reported themselves as Male Sex Worker (MSW) and Female Sex Worker (FSW) respectively. This risk behaviour did not reach the level of significance p-value >0.05. The three MSW were reluctant in giving further details of sexual activity and partners.

The use of injections both for therapeutic and drug intake purpose were significant risk factors for HIV infection (p-value 0.001).The study subjects with a history of use of injections for therapeutic reason had two times more risk of infection as compared to controls[OR 2.791, 95%CI (1.718-4.5340)].

Study participants who visited foreign countries for business purpose or as labour and those who deported were included in migrants. Statistical analysis showed that such people had more at risk of acquiring HIV infection [OR 2.234, 95 % CI (1.316-3.79)] as compared to non-migrants.

Tattooing was also observed as a risk factor with p-value 0.002. Similarly study subject with the previous history of blood transfusion were two times more prone to infection, i.e. [OR 2.69(95% CI 1.23-5.86)].

Medical history of study subjects including any surgical operation and major/minor dental treatment were observed not to be associated with HIV infection (p >0.05).Similarly, other non-sexual high-risk behaviours like sharing of shaving instruments, acupuncture were also found to be non-significantly associated with infection.

## DISCUSSION

Currently, the HIV/AIDS epidemic is established in Pakistan and there is a risk of an expanded outbreak in the country.^1^ The present investigation is being the first study reporting the significantly associated risk factors in the population of KP.

According to our study males are more prone to infection as compared to females. This finding is in compliance with other studies conducted in different regions in Pakistan.^12^,^13^ The ratio for male: female is 2:1 according to our study that is similar to the findings of a study conducted by Iqbal and Rehan.^14^

With regard to residence, we found no difference in rural and urban population. This is in contrast to findings of Mohammad et al. who reported urban residents as a significant factor of acquiring an infection.^15^ The observed difference is due to the difference in population density of the study area.

The state of being married/unmarried does not affect HIV infection in our study. This is in contrast to the findings of a study conducted in Faisalabad where HIV is more prevalent in widow/divorced individuals.^15^

Many studies were conducted regarding finding the predictors of unemployment in HIV positive patients.^16^,^17^ This is in contrast to our study where we found a high number of HIV patients being employed. HIV status is a social stigma in our country, and usually, most infected people do not disclose their disease status at their workplace.

The use of therapeutic injections is also another significant risk factor examined in our study. This finding is supported by a study conducted by Ahmad et al. that showed that 19% of spouses of IDUs sometimes received injection drugs along with their IDU husbands. They used the facilities of the same health care centre that provided therapeutic injections to the other members of the community using the same non-sterile injecting equipment. In this way they may, serve as a bridge for the spread of HIV from IDUs to the general population through the use of shared syringes.^18^ In our study, we found that IDUs are at high risk of acquiring infection. In Pakistan, since the last thirty years, the highest infection rate of HIV is observed in IDUs.^19^

Currently, Pakistan is facing the issue of migrants who are deported because of their HIV positive status. They are far away from their families for years that led them to involve in unsafe sexual practices. We identified the same as a significant factor. This fact is also highlighted by Shah SA et al. in Sindh.^20^

According to the present study, blood transfusion is significantly related to the acquiring of infection. In Pakistan, it’s a very common and popular form of treatment. Although there is a very low percentage of HIV in blood donors as reported by Aisha et al. but cases of HIV had been reported due to blood transfusion.^21^,^22^ A slow and continuous increases in the HIV-Positive cases among blood donors was reported from a study conducted in northern Pakistan.^23^

Tattooing has been recognized as a potential risk factor for HBV, but a little data is available on its role in HIV transmission.^24^ The same association was found in the Middle East and North Africa.^25^ Tattoing is also found in the history of prisoners a study conducted by Muhammad DK et al.^26^

In Pakistan, HIV is reported from MSW as according to studies conducted.^9^,^27^,^28^ A study conducted by Tahira et al. showed that among the major cities of Pakistan, Peshawar had the lowest prevalence of MSWs as well FSWs and the former one being more prone to infection.^29^ This is similar to the finding of our study where we reported no FSW and three Male Sex Workers (MSWs). An alarmingly higher risk of infection from the spouse with HIV is reported by our study that is in compliance with the findings of the study conducted in Tanzania.^30^

### Limitations of the study

The limitation of this study is the unavailability of transgender community.

### Conclusions

The present study revealed spouse status of HIV, tattooing, migrants, IDUs, use of therapeutic injections, history of blood transfusion, male gender and employment as significant risk factors for HIV infection in the population of KP.

### Recommendations

After the findings of the current study, we recommend the following preventive measures.

- Immediate Screening of family of HIV positive Individuals.
- Awareness in people regarding the different routes of HIV infection.
- Regular screening of migrants and IDUs.
- Taking strict actions for reuse of syringes in formal and non-formal health care facilities.
